# Whisper: Programmable and Flexible Control on Industrial IoT Networks

**DOI:** 10.3390/s18114048

**Published:** 2018-11-20

**Authors:** Esteban Municio, Johann Marquez-Barja, Steven Latré, Stefano Vissicchio

**Affiliations:** 1 IDLab—Department of Mathematics and Computer Science, University of Antwerp—IMEC, 2000 Antwerp, Belgium; steven.latre@uantwerpen.be; 2 IDLab—Faculty of Applied Engineering, University of Antwerp—IMEC, 2000 Antwerp, Belgium; johann.marquez-barja@uantwerpen.be; 3 University College London, WC1E 6BT London, UK; s.vissicchio@ucl.ac.uk

**Keywords:** SDN, Internet of Things (IoT), RPL, 6TiSCH, Whisper

## Abstract

Software Defined Networking (SDN) centralizes network control to improve network programmability and flexibility. Contrary to wired settings, it is unclear how to support SDN in low power and lossy networks like typical Internet of Things (IoT) ones. Challenges encompass providing reliable in-band connectivity between the centralized controller and out-of-range nodes, and coping with physical limitations of the highly resource-constrained IoT devices. In this work, we present Whisper, an enabler for SDN in low power and lossy networks. The centralized Whisper controller of a network remotely controls nodes’ forwarding and cell allocation. To do so, the controller sends carefully computed routing and scheduling messages that are fully compatible with the protocols run in the network. This mechanism ensures the best possible in-band connectivity between the controller and all network nodes, capitalizing on an interface which is already supported by network devices. Whisper’s internal algorithms further reduce the number of messages sent by the controller, to make the exerted control as lightweight as possible for the devices. Beyond detailing Whisper’s design, we discuss compelling use cases that Whisper unlocks, including rerouting around low-battery devices and providing runtime defense to jamming attacks. We also describe how to implement Whisper in current IoT open standards (RPL and 6TiSCH) without modifying IoT devices’ firmware. This shows that Whisper can implement an SDN-like control for distributed low power networks with no specific support for SDN, from legacy to next generation IoT devices. Our testbed experiments show that Whisper successfully controls the network in both the scheduling and routing plane, with significantly less overhead than other SDN-IoT solutions, no additional latency and no packet loss.

## 1. Introduction

Advanced network management is becoming crucial for Internet of Things (IoT) networks, especially since they are employed more and more in industrial deployments (Industrial Internet of Things). In these deployments, IoT devices typically mission control critical infrastructures, thus mandating high reliability, low latency, and careful energy consumption, which only dynamic and flexible network management can offer.

Software Defined Networking (SDN) is a promising architecture to enable flexible and dynamic network management, in a programmable and highly automated way. It achieves these goals by relying on a logically centralized controller that automatically configures all network devices, at runtime. SDN is already popular in wired networks, where it has been shown useful for a number of management tasks [1]. SDN concepts have also been applied to access points and base stations in infrastructure-based wireless networks, like Wi-Fi, WiMAX or LTE [2,3]. We argue that SDN can also be beneficial for IoT networks.

However, implementing SDN in Low-power and Lossy Networks (LLNs), such as the industrial IoT networks, is significantly challenging, much more than in wired networks. An LLN is usually organized in a wireless mesh, which implies the absence of out-of-band connectivity between the controller and the devices: control must therefore be exerted in-band, on a reliable communication channel over the lossy network. In addition, LLNs are highly constrained in terms of resources, both from a device and a network viewpoint: not only do the devices have low processor power, scarce memory, and limited battery life, but also network bandwidth and availability are constrained—for example, most devices can be in sleep mode during more than 99% of the time. This means that a practical SDN solution must minimize resource utilization for control tasks, e.g., keeping the number of control messages as low as possible and computations outside the devices. The latter observation also explains difficulties in designing SDN protocols for industrial IoT and integrating even a lightweight SDN stack on the devices. Finally, networks forwarding performance depends on both routing and scheduling functions, hence an IoT SDN controller needs to control both.

**Figure 1:**
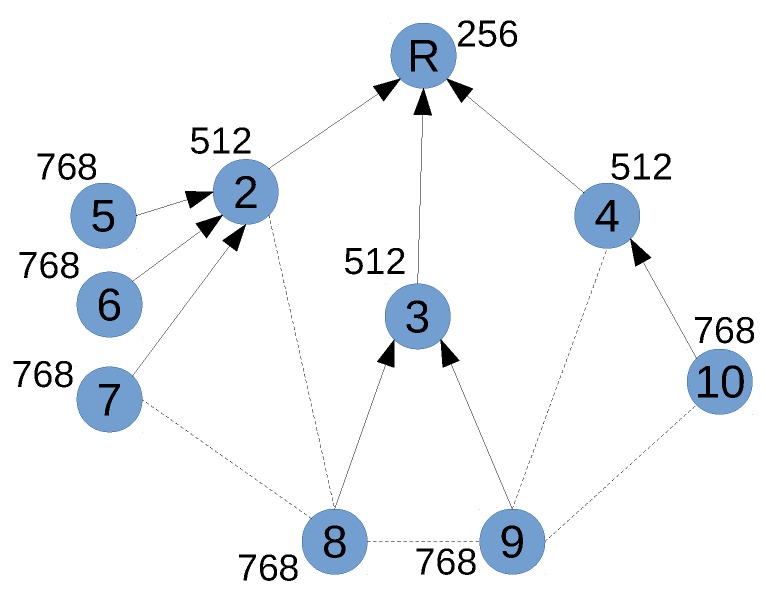
Example of RPL topology.

In this work, we propose Whisper, (Wireless Heterogeneous IoT SDN-enabling Protocol for Embedded and Resource constrained devices), a practical centralized management system for industrial IoT networks which enables a central controller to remotely manipulate routing, and scheduling of any network device. To address the SDN-in-IoT challenges, a Whisper controller leverages from the distributed routing and scheduling protocols already running in the network to both establish reliable communication and control remote devices. For example, assuming that Figure 1 represents a traditional LLN network in which nodes are wirelessly connected and build a tree to route traffic towards a gateway (node R) by means of a metric. Whisper can achieve specific dynamic (re)routing behaviours by artificially manipulating the metrics which define routing paths, thus managing the wireless resources accordingly. Such behaviours are not allowed by default in the routing protocol. For instance, by altering the routing metrics in the network to steer the traffic from nodes 8 and 9 through node 4, skipping node 3 due to low battery issues, and avoiding overloading node 2. The Whisper control mechanism is compatible with device resources, as the exploited protocols are run in the first place; and avoids the need for additional SDN-specific support on IoT devices, which also has the positive side effect of working in today’s networks. To further reduce resource usage, Whisper aims at minimizing the information injected in the underlying protocols.

In practice, a Whisper controller controls the IoT network by disseminating misleading information on purpose, or that enables the controller to “impersonate” other nodes. When receiving such messages, receivers are persuaded to change their next-hop and manage the wireless resources accordingly. We demonstrate that Whisper works on already established routing and scheduling protocols designed for IoT networks without modifying them neither adding additional software in the devices. Namely, our implementation works on top of the Routing Protocol for Low-power and lossy networks (RPL) [4] and 6TiSCH [5], an industrial IoT open standard for running IPv6 in the Timeslotted Channel Hopping (TSCH) mode of IEEE 802.15.4e (one vendor alone reports over 39,000 TSCH networks deployed, which is over 10-Billion operating hours [6]). While RPL are widely used, proprietary scheduling protocols may be run in industrial deployments instead of 6TiSCH. We strongly believe that Whisper can be easily extended to RPL based proprietary technologies, subject to syntactic changes in the messages sent by the Whisper controller.

We identify alternative message types, dissemination possibilities, and deployment options to implement Whisper centralized controllers. We design an algorithm for Whisper controllers to efficiently calculate the combination of those alternatives that reduces control overhead and device load the most. The result is that Whisper can operate by only deploying the controller at the RPL-tree root node. With only the controller in place, however, it is not always possible to arbitrarily control routing and scheduling at every node, even when technically possible (e.g., next-hops are in the wireless range of their respective previous hops). The Whisper-controller algorithm identifies those cases, indicates where to deploy new nodes (if needed), and optimizes their usage—e.g., to forward traffic between two consecutive hops on the desired path, or allow additional scheduling operations. We refer to such new nodes as Whisper nodes.

In building Whisper, we make the following contributions:

- We describe the basics of Whisper, and compelling use cases unlocked by our system (Section 3.1).
- We detail the design of the Whisper controllers, along with their internal algorithms to translate high-level policies into a limited number of control messages (Section 3.3 and Section 3.4).
- We pinpoint the limitations of only relying on a single Whisper controller, and show how the controller can systematically use Whisper Nodes (if deployed or deployable) to circumvent those limitations (Section 4).
- We report on our Whisper implementation (Section 5), and evaluate the effectiveness of our approach with simulations and experiments with real IoT devices (Section 6).
- We outline Whisper longer-term perspectives (Section 7).

## 2. Background

### 2.1. Whisper with Respect to Related Work

A huge amount of work has been done in the past on wireless sensor networks, aiming at improving both routing [7,8,9,10,11,12] and medium access [13,14,15,16,17,18] protocols. Prior contributions, however, overlooked the need for control flexibility, and focused on distributed protocols. What today’s IoT networks would really need, though, is a form of centralized control. In fact, centralized architectures have been proven effective in wired networks [19] to achieve optimal, dynamic control. We consider the question of what could be a practical centralized architecture for industrial IoT deployments. There are two main design possibilities: (i) adding support for an SDN-like protocol on top of MAC and network layers tailored to IoT (traditional approach), or (ii) integrate network control with current MAC and network layers (Whisper approach). The first option is the one on which SDN for IoT research is currently focusing [20,21,22]. We argue why this is not the only right way to go, and why integrating network control with medium access management and routing is inherently more promising.

SDN originated and spread quickly in wired networks [23], where it combines huge advantages (e.g., flexibility over an otherwise ossified environment) with relatively easy deployment (e.g., possibility to connect nodes and controllers out-of-band). Advancements in the field have led to complex management applications such as defining network slices, enforcing per-flow Quality of Service, and dynamically orchestrating virtual network functions. For an overview of such advancements, we refer the reader to [1,24] and the successful deployments presented in [25].

Among the works on SDN for wired networks, Fibbing [26,27] is the closest in philosophy to ours. In Fibbing, a centralized controller injects information about fake links and nodes in an underlying shortest-path routing protocol run by the real routers. The injected information enables the controller to control the output of routers’ computations, and ultimately their forwarding. Whisper shares with Fibbing the idea of injecting misleading messages in the routing plane. However, control mechanisms, algorithms, messages (format and content), and overall trade-offs are all entirely different. For example, to be practical in IoT settings, Whisper controls its wireless network without requiring nodes to compute shortest paths on the full network and without adding fake nodes or links which would increase the number of messages as well as the information kept by routers. In addition, Whisper addresses the peculiar needs of wireless mesh networks: for example, it controls the scheduling plane, coordinating cell allocations, in addition to routing.

SDN is relatively less popular in wireless networks [2,3,28,29]. SDN over wireless approaches mostly focuses on settings where management is performed by infrastructure-based access points and base stations of Wi-Fi, WiMAX and LTE. In these networks, protocols like OpenFlow [19] can be run on the access points, since they have sufficient hardware resources. Moreover, there are already well established management protocols for these types of networks.

In LLNs, however, devices are highly resource constrained, which makes it impossible to transpose SDN solutions from wired or other wireless environments. Only a few works focus on making the first steps towards introducing SDN in LLNs and IoT networks. Thubert et al. [30] presents the work of the DetNet [31] and 6TiSCH [32] IETF Working Groups (WG), and identifies the challenges faced by SDN in IoT networks—most of which we discussed in this paper. Constanzo et al. [33] describe a general architecture and discuss some of the constraints and requirements for SDN over IoT. A few SDN approaches for wireless sensor networks have been proposed in De Oliveira et al. (TinySDN) [34] and in Gallucio et al. (SDN-WISE) [20]. Contrary to Whisper, the above solutions however require support special-purpose configuration protocol on all network nodes, have a considerable resource footprint and require a direct and reliable out-of-band connection between nodes and the controller. These factors limit the realizability of these approaches in LLNs.

In addition, and in contrast with wired networks, IoT environments are becoming more and more heterogeneous, with multiple technologies that may overlap in spectrum usage and are managed in a completely independent fashion [35]. These technologies are deployed through embedded devices from multiple external vendors. While these devices often use standardized communication protocols (Constrained Application Protocol (CoAP), RPL, etc.), there is no often way of modifying the firmware of all intermediary routers to conform an SDN network. In that sense, Whisper provides control over all devices that implement basic open standards (RPL and 6TiSCH) for routing and cell allocation without modifying the firmware of the nodes in the network.

### 2.2. Background on LLN and IoT Protocols

LLNs and IoT networks often rely on the IEEE 802.154e TSCH mode for the MAC layer and the RPL protocol for routing. 6TiSCH is defined by the 6TiSCH IETF WG [32] with the goal of creating a standard protocol based on TSCH attached to an IPv6-enabled IoT upper stack: it aims at providing deterministic performance and ultra-relatability with low consumption of nodes’ resources [5,17,36].

The 6TiSCH stack is built upon the IEEE 802.15.4 PHY layer, which typically uses 16 orthogonal channels in the 2.4 GHz ISM band (in addition the standard allows the 868 MHz band). On top of this, the TSCH layer divides the channel access in timeslots of typically 10 ms duration that allows for sending, at 250 kbps, a 127 byte size packet and receives a short ACK. Timeslots are made of a number of grouped slotframes. The combination of timeslots in a slotframe and the available channels result in a Channel Distribution Usage (CDU) matrix, in which each cell is a combination of a timeslot and a channel offset.

A scheduler included in the 6Top Sublayer defines when a node has to transmit, receive or sleep during one of these cells. Starting from the Minimal Configuration [37], a node can build its schedule according to a Scheduling Function (SF) by using a set of Sixtop Protocol (6P) messages. The defined SF in 6TiSCH by default is the Minimal Scheduling Function (MSF), and allows each node to allocate or deallocate cells according to its traffic demands. In 6TiSCH, and in other TSCH-based solutions such as Orchestra [38], RPL is used for building uplink and downlink IPv6 routes.

RPL is a gradient based routing protocol in which the network is built as a Destination Oriented Directed Acyclic Graph (DODAG) and each node has a rank value that monotonically decreases as nodes are further from the DODAG root. For traffic forwarding, every node locally selects its successor in the DODAG, or parent, which is the next-hop that the node uses for sending uplink traffic towards the root. Each node selects its preferred parent as the neighbor with the lowest perceived rank. Node ranks are locally broadcasted through DODAG Information Object (DIO) messages.

One or more performance metrics, such as Expected Transmission Count (ETX) or latency, are translated into a rank value by the Objective Function (OF). The default objective function used by RPL nodes is called OF0 [39], and relies on ETX and hop count.

For downlink traffic, routing information is propagated through Destination Advertisement Object (DAO) messages. RPL has two operational modes. In the storing mode, every node maintains a routing table and the DAOs are sent from each child to its parent. In the non-storing mode, the exact path to follow is specified in the downlink-traffic packets: paths are calculated by the root, which receives DAO messages from all the nodes.

Consider again the network depicted in Figure 1, where the DODAG is represented as a set of arrows, from each node to its respective parent, and dotted segments identify neighbors with no parent relationship. The rank of any node, the number illustrated by the node, is calculated as the rank of its parent plus a value that depends on the quality of the link between that node and its parent (e.g., in OF0). For example, node 8 chooses node 3 (it could have chosen 2 as well) as its parent because it perceives with it a lower rank than with 7 or 9. After choosing a parent, the node keeps that parent until another neighbor offers a rank significantly better than the one of its current parent—where it is significantly quantified by a threshold (e.g., 640 in 6TiSCH by default).

Although the OF0 is defined as the default RPL OF, different OF have been proposed [40,41,42] to optimize different Quality of Service parameters by assigning link weights differently. In addition, non-standard “*forks*” of RPL have been explored [43,44,45]. Although enabling optimization of different metrics, these approaches do not really achieve network programmability and management flexibility. For example, to change the optimization criteria, the entire RPL layer has to be modified, requiring the replacement of all nodes’ firmware. This is both impractical and limited, e.g., for implementing prompt reaction to events or dynamic optimization.

## 3. Whisper Framework: SDN without SDN

### 3.1. Central IoT Control by Whisper-Ing

The Sixtop subayer describes the technical means for nodes to communicate with their neighbors, and RPL provides the basic information for forwarding packets. They both implement statically defined decisions—e.g., parent preference in RPL through a predefined objective function. Dynamics, however, are an important aspect of networks, especially IoT ones, where resources can suddenly deplete and network conditions change. This calls for a more flexible network management system. Whisper aims at realizing such a system while also exposing a high-level interface for operators to define their management objectives (programmability).

Whisper’s centralized controller (e.g., a server) is at the root of the RPL DODAG, or directly controls the messages received and sent by the root. The controller can therefore communicate with root’s neighbors, exchanging 6P and RPL messages with them; and it uses this possibility to craft messages that conveniently influence routing and scheduling decisions of nodes which are potentially far away. For example, by disseminating untruthful information about its rank to some of its neighbors, the controller can modify the RPL DODAG. Consider again Figure 1, and suppose that the Whisper controller positioned in R sends a DIO to 3 “whispering” that R’s rank is higher than 256. This will increase the rank of 3, which in turn will locally broadcast its new increased rank. Depending on the rank value whispered to 3, this single message can induce a subset of 3 and its children (8 and 9, in the example) to change their respective parents. Whisper exploits this mechanism for implementing high-level policies (see Section 3.3).

We now present a few illustrative examples of Whisper’s ability to implement practical policies.

   **Whispering for better routing**

**Figure 2:**
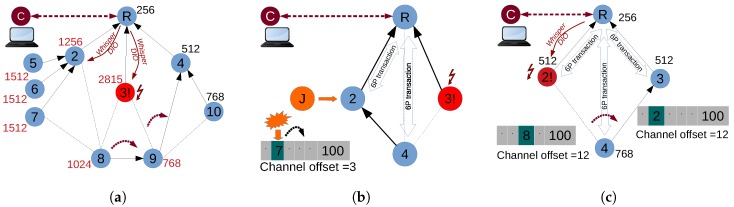
Whisper better supports dynamic management use cases, impossible to handle with only current IoT protocols. (**a**) rerouting around low-battery node while load balancing; (**b**) jamming defense; (**c**) smooth parent switching.

Currently, human operators and network management systems cannot dynamically adapt routing to unexpected events, such as depletion of the battery of a given network node. Consider again the example network in Figure 1, and suppose that node 3 starts running out of battery. The ideal response to this event would be to switch the parents of nodes 8 and 9, from 3 to 10 for node 9 and from 3 to 9 for node 8, as shown in Figure 2a. This would re-route traffic from 8 and 9 around the low-battery device (node 3) while also not overloading node 2 (which already has to carry traffic from nodes 5, 6 and 7). However, this is impossible to achieve in standard RPL networks. Some RPL OFs could optimize the routing in terms of energy and load balancing. However, these OFs are executed independently in different RPL instances, so that different traffic can follow different paths regarding how it is tagged. However, combining both OF would require developing another specific OF explicitly for this task. In contrast, we argue that by just using the default stand-alone RPL OF0 in a the network, Whisper can achieve the desired parent changes without modifying any bit in the firmware of the already deployed nodes.

Whisper achieves this behaviour by replacing standard root’s DIOs with periodic unicast fake DIOs with an artificial high rank (e.g., 2599) that will be propagated to nodes 8 and 9 through node 3. Due to this propagation, they will switch their parents to 2 and 4, respectively. In order to make 8 pick 9 as its preferred parent, the root can propagate as well through node 2 an artificial high rank (e.g., 1000). Both nodes 8 and 9 will keep their newly selected parents since the difference for changing back to other tentative parents is not large enough.

**Whispering for better scheduling**

Whisper can also be used for managing the scheduling plane. Consider the network in Figure 2b which runs 6TiSCH. Suppose that a malicious node starts a jamming attack that selectively interferes with the cell (7,3) between 2 and 4. If the interference persists (i.e., follows the channel hopping pattern), the ETX in the link will eventually be increased and will provoke an undesired switch from 2 to 3. Note that the same would happen if a non-malicious node causes many collisions due to an uncoupled scheduling. The Whisper controller can trigger 6P commands so as to allocate and deallocate cells between 2 and 4, and make the nodes use the cells with higher Packet Delivery Ratio (PDR), avoiding the parent switch.

Using the same primitive, Whisper can dynamically implement other policies: imposing different SFs for minimizing latency [46,47], improving the PDR of a link [48,49], reducing collisions [50] and so on. The Whisper controller complements the decentralized SFs installed in the nodes when they do not address operators’ requirements. We note that this is completely different from acting as a centralized scheduler (e.g., a DetNet PCE [31]).

**Whispering for combined better routing and scheduling**

Whisper can also jointly modify routing and scheduling, e.g., for allocating cells before rerouting. For example, given the topology in Figure 2c), packets will be dropped if node 4 suddenly switches the parent because scheduling functions (e.g., MSF, LLSF, etc.) do not normally allocate cells between nodes that are not parent-child (i.e., no cells between 3 and 4). During the sudden parent switch, node 4 will thus have to enqueue the incoming packets, dropping them when the queue gets filled. Only when the SF detects that no cells are allocated with the new parent is the cell added and the packets can flow again towards the root.

Whisper coordinates operations so that a cell is allocated before the switch occurs. In addition, the unused cells with the old parent are deallocated in order to release channel resources (there are still keep-alive messages being sent) and save power in nodes 2 and 4.

### 3.2. The Need for More Whisper Nodes

The previous examples show how Whisper addresses different management needs which are impossible to tackle simultaneously without flexible and dynamic network control. There are, however, cases where a single controller cannot always arbitrarily manipulate the routing and scheduling functions of all the nodes in the controlled network. For instance, if node 4 is out of a root’s wireless range in Figure 2b, the controller cannot directly communicate with 4, and hence cannot persuade 4 to change its cell. This is because of the physical limits of wireless ranges, the wireless impairments, and the local reach of 6P messages.

We provide the Whisper controller with the ability to compute possible extensions of the control infrastructure which would overcome its physical limitations. The Whisper controller can indicate to operators where to deploy special-purpose nodes that the controller can use to implement otherwise impossible policies. In Figure 2b, for example, the controller would suggest that a new node is deployed in the range of 4 so that this node can issue the 6P messages needed for 4 to replace the jammed cell with another one. This does not apply to nodes’ forwarding since RPL is above the network layer.

We refer to these special-purpose nodes as Whisper Nodes: they can be plain IoT devices with a modified firmware or wireless hardware-enhanced devices. When deployed, the controller will delegate specific management actions to the Whisper Nodes through application protocols such as CoAP [51].

The full set of control primitives for both RPL and 6P used by the Whisper controller is shown in Table 1. In the following, we describe the design of the Whisper controller, and how it computes which primitives to exploit and how, when it acts as a single node (Section 3.3). We then discuss topological conditions that make the presence of other Whisper Nodes necessary, and explain how the Whisper controller orchestrates such nodes (Section 4.2).

### 3.3. Architecture

**Figure 3:**
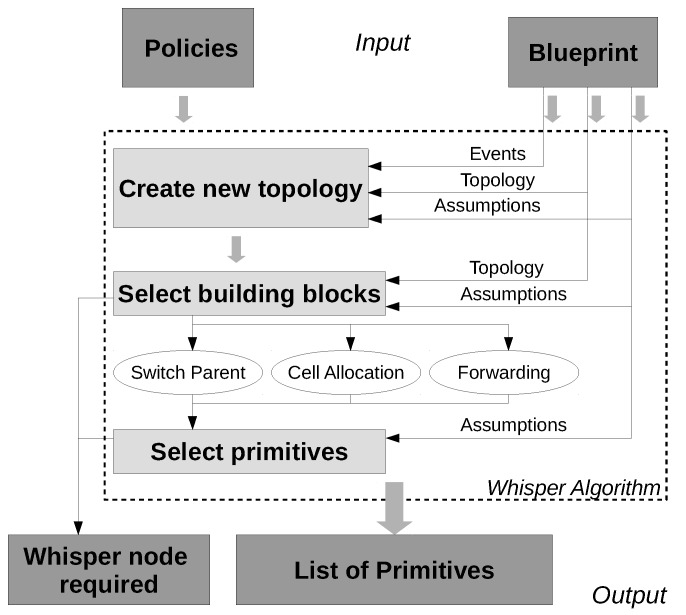
Whisper architecture.

The Whisper architecture is built as shown in Figure 3. It is based on three main components: policies, building blocks and primitives. First, some *policies* are given as input from the network operator. Example policies that can be applied are given in Table 2. These policies are applied to a given network blueprint, which is basically the network topology, the network assumptions and the events that can happen during operation. The assumptions state what type of capabilities the Whisper algorithm can have. For example, a number of assumptions can be made such as: Can we control the root node?, Can we place Whisper nodes anywhere?, Is MSF used as a scheduling function?, etc. Finally, the events are triggered at any instant of time in any node. These events can be triggered when a certain condition is matched in one of the nodes (Push), or can be periodically checked in the controller by polling the nodes (Pull). Whisper supports management at different time scales. Depending on the event and to which policy it is associated with, they can be processed in real time (e.g., DoS attack) or they can be aggregated and processed after a housekeeping period (e.g., load balancing).

By matching the policies to the blueprint, the Whisper algorithm first creates a logical structure of the newly desired (goal) topology. Depending on the policies and the differences obtained between the initial topology and the desired topology, the algorithm will generate the required Building blocks.

The different building blocks are the basic operations that can be performed in order to manage the network. There are three: *Switch parent*, *Allocate cell* and *Forwarding*.

***Switch parent***. This block is used whenever one or more policies require that a node changes its preferred parent. Every *Switch parent* block will trigger one or more primitives that will eventually end up in a parent change.

***Cell allocation*** (*and deallocation*). Through these blocks, Whisper can add or remove cells between any pair of network nodes. They can be applied together with the *Switch parent* block in order to allocate cells with the new parent and deallocate cells with the old parent, or can be applied alone, to perform scheduling-only operations.

***Forward traffic*** block is equivalent to the *Switch parent* block, with the only difference that the new parent of the target node is always a Whisper node that is part of the same DODAG. This way a Whisper node can attract the desired traffic towards him and relay it to another node if necessary. This can be useful for specific cases that require additional Whisper nodes in order to be accomplished such as policies that require using specific capabilities present in a Whisper node (e.g., block a suspicious Deny of Service attack through a DPI box located in the Whisper node). Additionally, due to the gradient nature of RPL, arbitrary topologies are not always possible. However by using this block, equivalent augmented topologies can be built with additional Whisper nodes in order to obtain specific routing behaviors.

Finally the last important component is the primitive. Each building block generates one or more primitives. Each primitive translates into a list of messages that have to be sent from a node X to node Y with a specific value as illustrated in Table 1. This value can be the rank for the DIOs or the sequence number SeqNum in the 6P commands. The Primitives 1–4 with DIOs messages are used to perform parent switching. Finally, Primitives 5 and 6 deliver fake 6P commands for just adding or deleting cells in the nodes’ schedule.

### 3.4. Algorithms Description

The three-step workflow illustrated in Figure 3 shows the three algorithms used in Whisper: FindNewTopology (Section 3.4.1), FindBlocks (Section 3.4.2) and CreatePrimitives (Section 3.4.3):

#### 3.4.1. Creating New Topology

This first algorithm creates a new topology derived and built from the initial one given as input according to the network blueprint and the policies to be applied. This procedure can be addressed either by an optimization algorithm or by an heuristic. In this work, we have chosen the heuristic since it renders the same solutions in significantly less computing time. This heuristic is a greedy procedure that basically consists in first identifying the triggering nodes $a_{i}$ (e.g., a node that have low battery), and afterwards, for every one of its children $ch_{a_{i}}$, select the next preferable parent $d_{t_{j}}$ as the preferred parent other than the triggering node. Finally, each of these topology changes will be rated according to the policy that triggers them and they will be checked to be loop-free. The more preferable solution will be selected and will be used in the next step of the algorithm as desired topology. This algorithm is described in Algorithm 1.

**Algorithm 1** Create new topology
1:
**procedure** FindNewTopology (givenPolicies, givenTopology)
2:    **for** policy in givenPolicies **do**  3:        **if** (policy== Policy 7 or policy== Policy 9) **then** continue4:        **for** $a_{i}$ in triggeringNodes **do**5:           **for** $t_{j}$ in $ch_{a_{i}}$ **do**  6:               selectNextCandidate of $t_{j}$, $d_{t_{j}}$7:               remove link $l(t_{j},a_{i})$  8:               add link $l(t_{j},d_{t_{j}})$  9:               save topology and $d_{t_{j}}$10:    **return** rateTopologies()


The policies that do not require a topology change and manage only the scheduling (e.g., Policies 7 and 9), will skip this step and will go directly to the next step.

#### 3.4.2. Selecting Building Blocks

Once the new topology is generated, the next step of Whisper will select the needed building blocks. This is a trivial step that will basically translate the differences between the initial and new topology to a list of building blocks regarding the assumptions. This means that the eventual result of applying one of the given policies will be one or more of the building blocks: *Switch parent*, *Cell Allocation* and *Forwarding*.

The algorithm, detailed in Algorithm 2, iterates for every link that differs between the old and the new topology and will add a new *Switch parent* block if no additional scheduling task is needed. Otherwise, *Allocate cell* and *Deallocate cell* blocks will also be needed in order to add cells with the new parent $d_{t_{j}}$ or delete cells with the old parent $c_{t_{j}}$. If the root is not in range to perform the scheduling blocks in the required nodes, the algorithm will notify the operator that the scheduling block cannot be performed. Additionally, if the parent $d_{t_{j}}$ is a $w_{i}$ node, the block to be used will be *Forwarding*.

**Algorithm 2** Select Building Blocks
1:**procedure**FindBlocks (givenTopology, newTopology)  2:    diff(givenTopology,newTopology)  3:    **for** $l(t_{j},d_{t_{j}})$ in sorted(diffLinks) **do**  4:        **if** $d_{y_{i}}$ in $w_{j}$ **then**  5:           **if** SF allows smooth switching or no smooth switching required **then**  6:               Add new *Forwarding* block: change parent of $t_{j}$ from $c_{t_{j}}$ to $w_{i}$7:           **else** 8:               Add new *Allocate cell* block: add cell between $t_{j}$ and $w_{i}$  9:               Add new *Forwarding* block: change parent of $t_{j}$ from $c_{t_{j}}$ to $w_{i}$  10:               Add new *Deallocate cell* block: delete cell between $t_{j}$ and $c_{t_{j}}$  11:        **else** 12:           **if** SF allows smooth switching or no smooth switching required **then**  13:               Add new *Switch parent* block: change parent of $t_{j}$ from $c_{t_{j}}$ to $d_{t_{j}}$14:           **else** 15:               **if** Whisper node or *r* in range of $\left(c_{t_{j}}\cap d_{t_{j}}\right)$ **then**  16:                   Add new *Allocate cell* block: add cell between $t_{j}$ and $d_{t_{j}}$  17:                   Add new *Switch parent* block: change parent of $t_{j}$ from $c_{t_{j}}$ to $d_{t_{j}}$  18:                   Add new *Deallocate cell* block: delete cell between $t_{j}$ and $c_{t_{j}}$  19:               **else** 20:                   Whisper node $w_{i}$ needed in the range of $\left(c_{t_{j}}\cap d_{t_{j}}\right)$→ create G*(N’,E’)  21:    Manage policies without routing changes if Policy 7 or Policy 9


#### 3.4.3. Selecting Primitives

Finally, the last step of Whisper translates each building block into one or more primitives. There are different algorithms to create primitives—one for each block—and they will be executed every time a block is needed. The most important algorithm is the one that creates the primitives for the *Switch parent* block which we detailed in Algorithm 3 (the algorithm for the *Cell allocation* block is only a simple translation to 6P commands (add or delete). The Forwarding block is a particular case of *Switch parent* where $d_{t_{j}}=w_{i}$).

Algorithm 3 iterates through the parent candidates $PC_{t}$, of the target node *t* that has a tentative rank lower or equal to the rank the target node perceives from its desired parent $d_{t}$. For every of these parent candidates, it checks if they are in the same branch as $d_{t}$. In case they are in the same branch, only if the node is not in the same branch as *t* as well and there is only one other neighbor with the same rank can the solution be done in three steps. First, one switches towards the neighbor with the lowest rank with a Primitive 1, the second increases the rank of the branch of t with a Primitive 2 and performs a second switch towards $d_{t}$ with an extra Primitive 1. Otherwise, the algorithm will notify the operator that there is no solution with the current assumptions and that a Whisper node has to be used.

In case the parent candidate is not in the same branch as $d_{t}$, then the rank of the branch will be increased directly from the root by sending a propagating DIO (Primitive 2) to the head of the branch of that candidate. This way, by artificially increasing the rank of the different candidates directly from the root, the rank that t sees from $d_{t}$ will be the lowest. This way $d_{t}$ will be chosen by *t* as preferred parent after receiving a remote DIO (Primitive 1) from the root with an artificial rank increase *inc*. However, these increments will not be always possible, since they could trigger undesired additional parent switches in other parts of the branch, if not a cascade effect that could destabilize the entire network. In order to know if these increments are possible, the so-called constraint nodes $CN_{t}$ are obtained for a given increment *inc*. These constraint nodes are the ones that would provoke an undesired switch if they receive an increment of rank *inc*. Once they are obtained, the function *isPossibleExtendDioPropagation()*, following an iterative greedy approach, calculates if there can be increments of *inc* in the branches coupled with the constraint nodes.

**Algorithm 3** Select Primitives: Switch Parent block
1:
**procedure** SelectPrimitives()
2:    **for** *n* in $PC_{t}-\left\{d_{t}\right\}$ if $RANK_{t}\left(n\right)<=RANK_{t}\left(d_{t}\right)$ **do**  3:        **if** ($P{\left[0\right]}_{n}$ == $P{\left[0\right]}_{d_{t}}$) **then**  4:           **if** $P_{n}\left[0\right]$ == $P_{t}\left[0\right]$ **then**  5:               A W node is needed. Abort and report with new G*(N’,E’) **return**  6:           **else** 7:               **if** Len ([for m in ($PC_{t}-\left\{d_{t}\right\}$) if $P_{m}\left[0\right]=P_{n}\left[0\right]$])=1 **then**  8:                   get $CN_{t}\left[I\right]$  9:                   $inc=abs(RANK_{t}\left(d_{t}\right)-RANK_{t}\left(n\right))+1$  10:                   **if** possibleExtendDioPropagation(inc) **then**  11:                       $I[P_{t}\left[0\right]]=inc$, if lower or equal than previous $I[P_{t}\left[0\right]]$  12:                       needExtraRemotePrimitive1=TRUE  13:                   **else** 14:                       A W node is needed. Abort and report with new G*(N’,E’) **return**15:               **else** 16:                   A W node is needed. Abort and report with new G*(N’,E’) **return**  17:        **else** 18:           get $CN_{n}\left[I\right]$19:           $inc=abs(RANK_{t}\left(d_{t}\right)-RANK_{t}\left(n\right))+1$  20:           **if** possibleExtendDioPropagation(inc) **then**  21:               $I[P_{n}\left[0\right]]=inc$, if lower or equal than previous $I[P_{n}\left[0\right]]$  22:           **else** 23:               A W node is needed. Abort and report with new G*(N’,E’) **return**24:        numNeighboursProcessed+=1  25:    **if** numNeighboursProcessed == 0 **then**  26:        **return** “Primitive 1: remote DIO from *r* to *t* with RANK 2599”  27:    **else** 28:        **return** preparePrimitives(*I*, needExtraRemotePrimitive1)


If these new branches have additional CNs, these new CNs will also be iterated until a stable solution is found, or until the entire network has been tracked. We discuss these special cases in Section 4.1. Finally, the end of the algorithm finishes preparing and ordering the primitives that the controller has to execute.

## 4. Coordination of Whisper Nodes

We have already mentioned that physical limits may prevent full control of the network from a single Whisper controller. We now describe cases when this occurs and how to deal with them within Whisper.

### 4.1. Augmenting Network Topology with Whisper Nodes

Additional Whisper Nodes are needed in the following cases:

**Per-node schedule reconfigurations**: 

The TSCH schedule can have a strong impact on the performance of the network. Changing slot schedules can lead to reduced latency, improved reliability, etc. As will be detailed in Section 5, Whisper modifies the TSCH schedule by injecting fake 6P commands, which allocate or deallocate slots between nodes. However, as the 6P protocol only has a local reach (no IPv6 header), additional Whisper nodes are required to also adapt the scheduling. In that case, the centralized controller will forward the request for specific slot changes to one or more whisper nodes. 

**Complex routing changes:**

**Figure 4:**
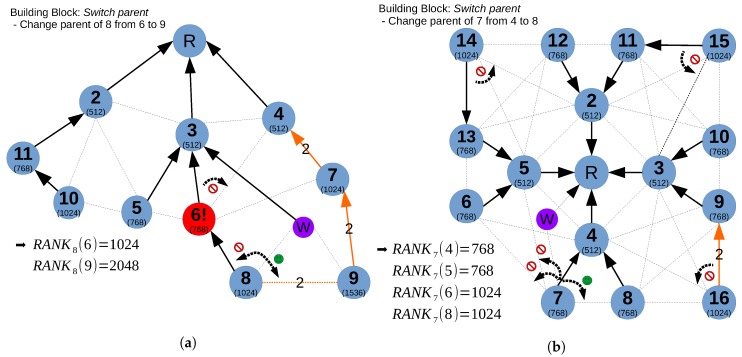
Example of two corner cases in which Whisper nodes are needed. Weights represent the ETX (ETX = 1 by default). (**a**) Example 1; (**b**) Example 2.

Figure 4 shows two examples where a single centralized controller cannot perform a specific routing change. In Example 1 in Figure 4a, the policies to accomplish are Energy and Load balancing. This means that when node 6 sends a notification of low battery, its children 8 should try to switch to the next parent candidate, i.e., 9. In this case, Algorithm 3 would report that a Whisper node is required since the parent switch of node 8 could not be done remotely from the controller without undesired parent changes such as the one of 6 from node 3 towards node 4. This is because the perceived rank difference in 8 between 6 and 9 is so large that an eventual increase in the branch of 3 used for stabilizing the switch (i.e., overcome the hysteresis threshold to avoid switching back) would force 6 to change its parent to 4, and therefore change its branch (which may not be interesting in terms of load balancing). If a W node is present, it could, by using a Primitive 4, relay node 8’s traffic through W towards 9 without altering the rank in the branch of 3.

Another example in which Whisper needs a Whisper node to accomplish the given policies is shown in Figure 4b. In this Example 2, for any reason, e.g., sensor data from 7 has to be processed in 8 for data fusion, the controller will determine that 7 should switch from 4 to 8. However, in order to achieve this, Algorithm 3 will try to increase the branch of 5 with a Primitive 2, followed by a Primitive 1 towards node 7. Since the branch of node 5 has node 14 as constraint node, it will try to increase the branch of 2 as well, which is linked at the same time with branch 3 through another constraint node (i.e., node 15). However, if the algorithm tries to increase branch 3, it will realize that the original branch 4 is coupled with branch 3 through node 16 (with a ETX = 2 link). For this reason, the algorithm will notify that a Whisper node is needed. In this case, a Whisper node could force the desired switch by impersonating 6,5 and 8 using Primitive 3.

### 4.2. Adding and Managing Whisper Nodes

By running the algorithms described in Section 3.4, the controller identifies the policies that it cannot realize, and outputs an error along with the nodes that are out of its reach. Based on this output, the operator can deploy new Whisper nodes. Suppose that the operator does so, and provides information about the new nodes to the Whisper controller. The controller will then re-run its algorithms to attempt to satisfy all its input policies again—successfully this time, if the Whisper nodes are deployed in the right places.

The Whisper controller can use Whisper nodes to either send control-plane (RPL or 6P) messages on its behalf, or to receive and relay traffic. In any case, Whisper uses such nodes only if necessary; in other words, it always prioritizes solutions using only Primitives 1 and 2 in order to minimize the generated overhead.

## 5. Whisper Specifications

We here describe the details of Whisper, including the message fabrication and other considerations.

### 5.1. Injecting Fake DIOs

The fake DIO injection is one of the key procedures in Whisper. In RPL, there is an hysteresis phenomenon introduced by the threshold value T used in order to make links more stable. Whisper leverages from this threshold in order to create an stable parent switch. By sending a DIO with a higher or lower artificial rank, the target node *t* can switch to the desired parent. Due to this hysteresis, only 1 DIO will suffice since, although after some time node *t* will receive a legitimate DIO from the old parent, *t* will not switch back due to the rank difference not being high enough. This is the concept that is behind the four available DIO primitives:

- **Primitive 1:** Fake DIO sent in unicast from the root to the *t* node remotely. It is considered as a normal DATA IPv6 packet in order to reach the remote destination, so it has the local MAC destination address of the next hop and the IP destination address of the remote node *t*. This type of fake DIO can only be used for supplanting the previous parent of *t*, but it has the advantage of being able to remotely trigger a parent switch in a node several hops away with only one message and without the need of modifying the rank of an entire branch, controlling the root node or using a Whisper node.
- **Primitive 2:** Fake DIO sent periodically in unicast from the root (MAC and IP source address) to the first node of the branch of *t* (MAC and IP destination address). When these types of DIOs are present, all DIOs sent from the root are sent in unicast, customizing the rank that each branch needs to have and replacing the normal DIOs sent in broadcast. Since these DIOs are sent periodically, the value of the rank in that branch will be constantly updated and propagated along the branch. Although this primitive is normally used in combination with Primitive 1 to make $d_{t}$ the most preferable parent, it can also trigger parent switches by itself.
- **Primitive 3:** Fake DIO sent in unicast from a Whisper node to *t* node locally. This DIO has the MAC and IP source address of the node that the Whisper node is supplanting and the MAC and IP destination address of the *t* node. This primitive can also be used for both switching a parent or in combination with Primitive 1. It is used for finding feasible solutions for some cases such as Example 2 in Figure 4.
- **Primitive 4:** Fake DIO sent in unicast from the Whisper node to the local *t* node periodically, the Whisper node being the desired parent of *t*. This type of DIO is equivalent to Primitive 2, with a Whisper node instead. This primitive is used when it is necessary to announce the presence of a Whisper node enabled for active traffic forwarding in the network.

The DIOs have to be consistent with the DODAG version used in the current DODAG instance, so that when changes in the topology happen, DODAG versions become updated as well. We address this by having always at least one node controlled by whisper being an active part of the network, either the root node or a Whisper node, for tracking the DODAG version.

Unlike standard DIO messages, our “well-crafted” Whisper DIOs are sent in unicast. This allows for reaching any node in the network that is part of the DODAG and not interfering in the Trickle Algorithm [52] in nodes other than *t*. In addition, since they are sent in unicast, IEEE 802.15e specifies that their frames have to be acknowledged (*Ack Request* bit to 1). This provides Whisper with more reliable control since it allows for re-transmitting fake DIOs in case of failure.

Finally, depending on how lossy the network is, the increments in the primitives can be complemented with a rank margin value M that would make the network more stable under ETX fluctuations on the links.

**Figure 5:**
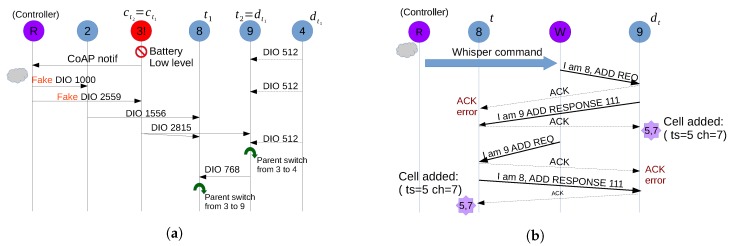
Example of sequence of messages for a parent switch and a 6P cell allocation. (**a**) parent switch for use case 1 in Figure 2a); (**b**) cell allocation for Example 1 in Figure 4.

In order to show how the process of the parent switch happens through fake DIOs, we illustrate in Figure 5a a sequence diagram of the first use case showed in Figure 2a and explained in Section 3.1.

### 5.2. Injecting Fake 6P Commands

6P commands do not have an IPv6 address, and hence they have a local reach and cannot be issued using multi-hop. One fake 6P transaction consists of issuing two fake 6P commands by the Whisper node each time (or by the root). The process of completing a 6P ADD transaction between two nodes by a Whisper node is illustrated in Figure 5b. The sequence shows the case in Example 1 of Figure 4 where nodes 8 and 9 would require having a cell (e.g., *cell (5,7)*) beforehand between them in order to provide a reliable parent switch and not lose packets.

First, upon receiving the primitive (i.e., Primitive 6), W sends a fake 6P ADD request to $d_{t}$ containing the desired Cell Options (slot, channel offset and direction) with the correct SeqNum. This fake 6P ADD request sent by *W* contains the MAC addresses of *t* as source address and $d_{t}$ as a destination address for a correct impersonation of *t*. On receiving this fake message, $d_{t}$ acknowledges the packet towards *t* and enqueues a new 6P ADD response with a destination also to *t*. As a consequence, *t* will ignore the ACK since it was not expecting any one. However, *W* will listen to the ACK to confirm that the transactions have been successfully initiated.

After $d_{t}$ manages to deliver the ADD response to *t* (either in the minimal cell or in a dedicated cell), *t* receives the packet and acknowledges it, but, when processing it in the sixtop sublayer, the packet will be discarded and ignored since *t* does not expect any response (is in IDLE state). However, $d_{t}$ will receive an ACK, which will trigger the allocation of the desired cell in the $d_{t}$ link side. The same happens for allocating a cell in the *t* link side. Since in MSF dedicated cells are SHARED (Cell Options 111) between the ends of the links, there is no need to reverse the direction.

Regarding the SeqNum, we assume that it is not known initially by the Whisper system. Therefore, Whisper tracks the SeqNum values used in each pair of links (e.g., with Whisper nodes). If not possible, fake 6P Commands can be issued with a SeqNum = 0. This will trigger a 6P Response with the response code RC_SEQNUM and will reset the SeqNum of the nodes to 0 making future 6P Command can be successfully executed, starting now with SeqNum = 1.

Finally, the Minimal Configuration specifies that 6P messages have to be sent through the minimal cell only when no dedicated cells exist between two nodes. This can be problematic for deleting cells with the 6P DELETE/CLEAR commands. Although the 6P request can be sent in the minimal cell, the 6P DELETE/CLEAR response will be sent back through one of the already existing dedicated cells, and the two-step process will end in only having deleted the cells on one side of the link. Whisper addresses this by issuing the 6P DELETE/CLEAR commands in the dedicated cells and ACKing when necessary.

### 5.3. Implementation Details

There are some assumptions that need to be addressed, such as knowing the current schedule in the network, the rank in the nodes and the neighbor topology.

In Whisper, we consider that there are different ways to obtain this information. The first one is using a management interface that allows the controller to poll the different nodes periodically for obtaining their rank values, their schedules and their neighbor topology. Consequently, this should be done through a standard interface such as YANG/NETCONF [53,54] for RPL, which is compatible with any RPL implementation.

In case these options are not available, another possibility is to obtain the information directly from sniffing, through some of the available tools for monitoring and diagnosing 6LoWPAN networks [55,56,57], which can be installed e.g., in the Whisper nodes.

For the case of the rank, this can be easily done by listening the DIOs in the minimal cell, requesting them with DODAG Information Solicitation (DIS) messages if necessary. In the worst case scenario, Whisper can still be valid for some use cases even without knowing the rank, since some parent switches can be performed simply by increasing the DIO discretely for some iterations until the parent switch is achieved. The same would happen for the scheduling, since the Whisper nodes can track which cells are allocated/deallocated by listening to the different 6P commands. For obtaining neighbor relationships (which are not included in the DODAG built in the root), it is possible to run in the Whisper nodes a neighbor discovering protocols existing for 6LoWPAN networks [58,59,60], or predict the physical location between the nodes through Received Signal Strength Indication (RSSI) analysis and signal triangulation [61,62,63].

## 6. Evaluation

We experimented with our proof-of-concept Whisper network in both testbeds and simulations. In the following, we describe the evaluation setup (Section 6.1) and the results (Section 6.2).

### 6.1. Testing Environment

For testbeds, we have deployed two 6TiSCH networks using OpenMotes. The OpenMote nodes consist in an OpenMote-CC2538 attached to an OpenUSB [64]. They run OpenWSN, using a variation of the release 1.14.0 enabling the implementation of MSF (available at [65]). As Whisper nodes we have used OpenMotes as well. The Whisper controller executes the Whisper workflow and communicates with the Whisper node through CoAP. This means that, in our implementation, Whisper nodes behave as normal nodes with the only difference that they issue fake messages under the reception of primitives and do not have children nodes. For monitoring the network, we run a modified version of Foren6 citedawans2014demo that tracks DIOs, DAOs and 6P messages (all Whisper software used is available at: [66]).

The goal of our testbed is to simply show that Whisper can control both scheduling and routing, without modifying the firmware of current IoT devices. We configured nodes to send uplink periodic 80-byte-packet User Datagram Protocol (UDP) traffic to the root node, and monitor packet loss and latency. In the Whisper nodes, an application is installed for translating each primitive into its corresponding fake message.

In order to compare Whisper with other IoT-SDN solutions, we have selected IT-SDN [67] (an updated version of TinySDN) and SDN-WISE [20]. These solutions have an implementation for ContikiOS [68] and are run in the Cooja emulator [69] with Sky Tmotes [70].

### 6.2. Results

**Figure 6:**
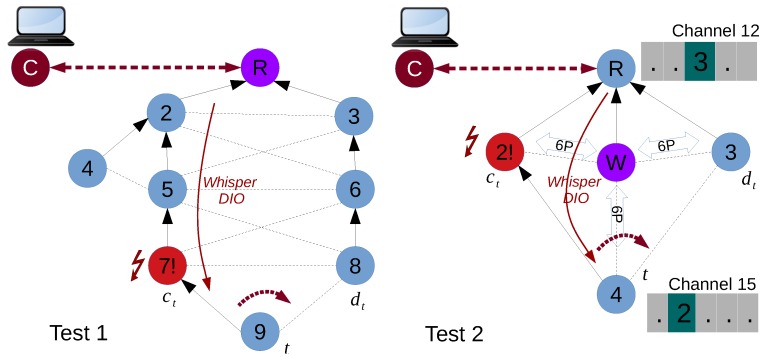
Topologies used for the two test use cases.

We considered the two cases shown in Figure 6. The set of policies and resulting primitives given by the Whisper algorithm for each test are shown in Table 3.

**Figure 7:**
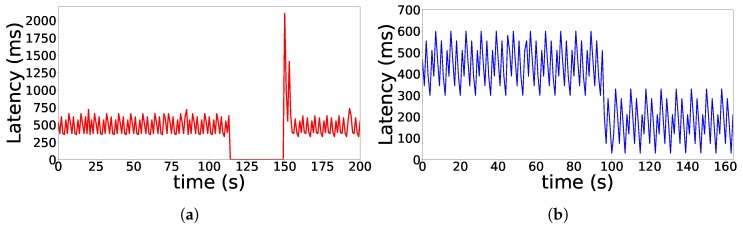
Latency when *t* changes its parent. (**a**) Test 1; (**b**) Test 2.

In both Test 1 and Test 2, the Whisper controller manages the root. In Test 1 (Figure 6 Test 1), the parent switch in the figure is triggered by sending a fake remote DIO (Primitive 1) from the root to node 9. Since there are no reliability or latency requirements, we allow the SF (which in our implementation is MSF) to perform the needed cell allocations and deallocations. Test 2 (Figure 6 Test 2) exemplifies a network where the Whisper controller uses a Whisper node *W* to actually exert network control. In fact, the controller instructs *W* to send fake DIOs type 1 to node 4, impersonating 2. Since latency and reliability are given as policies to implement, the *W* node performs the 6P allocations between 4 and 3 before the switch occurs and selects the cells that result in a lower end-to-end latency. After the switch, the cells between 4 and 2 are cleared. For both Test 1 and Test 2, results of end-to-end latency and packet loss before and after the parent switch are shown in Figure 7a,b.

For Test 1, Figure 7a shows packet drop and a peak in latency after the switch happens since cells between 9 and 8 are not allocated before the parent switch. Only after a housekeeping period, MSF triggers the 6P ADD transaction from 9 towards its new parent 8, and packet forwarding is re-established. After the switch, latency values are equivalent since MSF, which has a random cell selection process, has picked a cell that renders equivalent end-to-end latency.

Figure 7b illustrates that there is no packet drop and latency is lower after the parent switch in Test 2. This is expected since *W* allocates cells between 4 and 3 before the parent switch, making the transition occur smoothly. In addition, since the newly allocated cell (timeslot 2) is deliberately placed just before the cell between the root and node 3 (timeslot 3), the latency after the parent switch is lower.

To analyze the overhead introduced by Whisper in comparison with other centralized solutions, we compare it with IT-SDN and SDN-WISE using the Cooja emulator in the same topologies. In order to change the path from the target node to the sink, Whisper needs to receive the notification and issue only one Fake DIO in unicast to the target node (one Primitive 1). In contrast, IT-SDN and SDN-WISE need to send a significantly higher number of control messages in order to perform the same path change. Since SDN-WISE uses an OpenPath message to set up or update the flow table (in RSVP-fashion), it only requires sending three control packets: one for setting up the initial flow table, one for updating the flow table with the new path and the other to update the update the flow table in the nodes of the old path [67].

**Figure 8:**
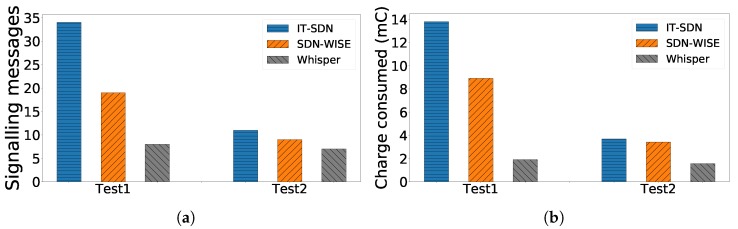
Overall signalling messages sent and charge consumed only for signalling related with routing in each of the compared solutions.

On the other hand, IT-SDN requires one control message per node in the path. This means that it will require:

(1) numMessages=numHops·(numHops+1)

on average. The number of messages of each solutions is shown in Figure 8a as the overall messages transmitted in both Test 1 and Test 2. For both SDN-WISE and IT-SDN, it is not possible to apply policies related with the scheduling (e.g., reliability, latency, etc.) since they cannot manage the MAC layer. For this reason, only routing related messages are considered for the three technologies.

In order to give an overview of the meaning of this overhead, we translate these values to charge consumed in the node’s batteries (without counting the root). For Whisper, we use an accurate 6TiSCH energy model [71] to calculate the energy consumed for both sending (139.57 μC) and receiving (152.97 μC) a packet in each node. For Contiki, we use simulations (2000 samples) to obtain the average consumption per packet for transmitting (419.12 μC) and receiving (130.1 μC) a packet. In order to illustrate these figures, each solution would consume the equivalent of two AA batteries (2000 mAh) only in signalling traffic in around 1 year (IT-SDN), 1.5 days (SDN-WISE) and 7.2 years (Whisper) when performing one switch as that described in Test 1 per minute.

Finally, in order to analyze the scalability of Whisper’s algorithms, we also simulate its application in a number of random topologies, with size ranging from 10 to 200. The networks are built and classified in two groups, one group “*sparse networks*” in which each node has at least one stable neighbor (PDR > 0.86) and a second group “*dense networks*” with at least three stable neighbors. This means that the “*sparse networks*” have a higher average hop count than the “*dense networks*” for all network sizes. Consequently, the average number of neighbors per node in the “*dense networks*” is higher. For every size, 100 different networks are tested; for every network, the Whisper’s algorithms are run 10,000 times. In each test, we ask Whisper to change the parent of a randomly picked node from its current parent to a randomly picked new one.

Results in Table 4 and Table 5 show that switches can be performed without the need of a Whisper node in at least the 50% of all the cases considered. This percentage varies regarding the size of the network and its density. The smaller and denser the network, the less probable it is that a Whisper node is needed in order to perform the switch. On the other hand, larger networks with less density are more prone to needing a Whisper node. This is because the parent switches are most effectively performed when nodes have a higher number of neighbors since Whisper can better control the rank perceived in the target node.

However, succeeding in triggering the parent switches without Whisper nodes comes at the cost of the signaling. The number of Primitives issued by Whisper per switch is between 1.5 and 3.8 on average in the *sparse networks* and between 2.8 and 10 in the *dense networks*. This means that there is a trade-off between not using Whisper nodes (and hence having a higher signaling overhead) and using them (and reducing the Whisper signaling).

The percentage of Primitive 1 messages are higher than Primitive 2 ones for small network sizes, and vice versa for larger networks. This is because the larger the network, the higher the coupling is between branches, so that more Primitive 2s have to be issued in order to enforce or stabilize the switch triggered by a Primitive 1. Finally, we note that the execution time of the algorithm is from a few microseconds to a few milliseconds for networks lower than 200 nodes. Higher computing times are expected in denser scenarios since more stack operations have to performed for identifying the constrain nodes. As a side note, computing time can vary significantly if optimization algorithms are used to calculate the primitives.

## 7. Whisper Roadmap

Our current Whisper system implements a precise design choice, that is, to exert centralized control compatible with (more precisely, through) *unmodified open-standard distributed protocols*. Throughout the paper, we argued for the several advantages of this design choice for SDN in IoT networks: pros range from compliance with both per-device and network-wide resource limitations to ease of deployment.

We are aware that Whisper’s design choice also has drawbacks. One of them is the inability of Whisper to cope with arbitrary levels of granularity—e.g., to control routing at a per-flow or at a per-packet level, as enabled by SDN protocols in wired networks [19,72,73]. Although RPL limits the use of differentiated services and traffic engineering techniques, there is still an open research space to exploit them by using different RPL instances [74]. Whisper’s full support for the use cases discussed in Section 3.1, however, suggests that this limitation might not be so critical in many practical SDN-over-IoT applications. In other words, Whisper might both leverage and demonstrate that the level of expressivity of IoT routing and scheduling protocols can be sufficient for SDN in IoT.

Nevertheless, we also contemplate the possibility that future research might find new use cases for SDN in IoT networks. We believe that the overall Whisper’s architecture might be useful in such cases. For example, our system can be easily adapted to *support* new SDN-specific protocols or enablers. While technically not full SDN, the research community is currently investigating several management interfaces (e.g., COMI [75], LWM2M [76]), network operating systems (e.g., ONOS [77]) and configuration languages (e.g., YANG [53]) for enabling management and control on top of LLNs. One example of this is shown in Anadiotis et al. [78], where the feasibility of evolving ONOS for supporting SDN-WISE is proved. Many other solutions often focus on application oriented configuration, while Whisper focuses on the network management aspects. However, we do not see Whisper as a competitor of these solutions but rather as a complementary solution. Several of the commands of those languages can be expressed through Whisper as well via in-band signaling. As such, Whisper can provide a solution for enabling COMI-based management on top of legacy networks. Future research will therefore be focused on integrating Whisper with such management solutions. An evolved Whisper can as such piggyback SDN protocol messages in RPL/6P messages, and hence offer a reliable and overhead-effective substrate for delivering finer-grained commands.

Finally, Whisper roadmap shares with SDN some of the open research issues identified in [79]. Two of the most promising are the development of schemes for efficient and reliable network information updates in real time and the coordination of multiple controllers in multi-domain SDN networks. In addition, future works can be oriented towards efficient dynamic load-balancing schemes and towards fast and cost-efficient failure recovery methods.

## 8. Conclusions

In this paper, we presented Whisper, a management system to centrally control low power and lossy networks, like IoT ones. To accommodate requirements stemming from this network setting, Whisper capitalizes on distributed routing and scheduling protocols run on the nodes. We described the design and implementation of our Whisper prototype, which is fully compatible with RPL and 6TiSCH: two popular open standards for IoT networks. We experimented with our implementation in a testbed with real IoT devices, showing that Whisper has promising performance in practice and lower overhead than other SDN-in-IoT solutions.

While its basic mechanisms might be effectively combined with future management protocols, we believe that Whisper can be a long-term complementary solution for flexible, SDN-like management of resource-constrained networks, where minimal overhead and support for heterogeneity are more critical than extreme (e.g., per-flow) control granularity.

## Figures and Tables

**Table 1:** Available primitives that determine the “*fake*” messages to be sent. W means Whisper node and R means root node.

1	Send remote DIO from node R in unicast to remote node X with rank RANK
2	Send propagating DIO from node R in unicast to local node X with rank RANK
3	Send supplanting DIO from node W in unicast to node X supplanting Y with rank RANK
4	Send propagating DIO from node W in unicast to node X with rank RANK
5	Send 6P ADD Req from node R/W to node X supplanting Y with seqNum SQ
6	Send 6P DEL Req from node R/W to node X supplanting Y with seqNum SQ

**Table 2:** Table with example of policies that can be applied. T means Threshold.

Name	Description	Rule	Push	Polling
Energy	Minimize traffic in a node that has a low battery level	if Battery < T r	x	x
Temp. (CPU)	Minimize traffic in a node that has high CPU temperature	if Temp. C${}^{\circ }$ > T	x	x
Queue (Memory)	Minimize traffic in a node that has high queue usage level	if avgQueue > T	x	x
Load Balancing	Maximize fairness of the routed traffic between the nodes	if loadNode1 > T + loadNode2		x
Deny of Service	Minimize effects of a DoS attack	if pkt/s > T	x	x
Reliability	Maximize end-to-end reliability	if e2eETXpath1 > T + e2eETXpath		x
Latency	Minimize end-to-end latency	if delay(path1) > T + delay(path2)		x
P2P traffic	Minimize hop count between two nodes	if p2p-path1 ∃	x	x
Mobility	Minimize packet loss in a parent switch	if RSSI-neigh1 > T + RSSI-neigh2	x	
Link PDR	Maximize PDR in a given link between two nodes	if cellPDR < T		x

**Table 3:** Table showing for each test the resulting primitives when applying the Whisper algorithm.

Network Blueprint	Policies	Building Blocks
Test 1	Energy, Load balancing	Switch parent
Test 2	Energy, Latency and Reliability	Allocate cell, Switch parent, Deallocate cell
Output Test 1	Primitive 1: Send DIO from node R to remote node 9 with RANK 2559	
Output Test 2	Primitive 5: Send 6P ADD Req from *W* to node 3 supplanting 4 with SeqNum 0	
	Primitive 5: Send 6P ADD Req from *W* to node 4 supplanting 3 with SeqNum 0	
	Primitive 3: Send DIO from node from *W* to node 4 supplanting 2 with RANK 2559	
	Primitive 6: Send 6P DEL Req from *W* to node 4 supplanting 2 with SeqNum 1	
	Primitive 6: Send 6P DEL Req from *W* to node 2 supplanting 4 with SeqNum 1	

**Table 4:** Results of running the algorithm for different network sizes in the “*sparse networks*” group.

Network Size (# nodes)	10	25	50	75	100	125	150	175	200
Avg # Hops	1.75	2.18	2.43	2.63	2.77	2.87	2.91	3.02	3.11
Avg # Neigh per node	3.58	5.55	7.65	8.99	10.13	10.63	11.73	12.34	12.88
Without W nodes (%)	93.6	83.9	75.0	65.4	61.2	57.7	54.0	52.1	50.1
Primitives per Switch	1.55	2.26	2.88	3.14	3.43	3.46	3.72	3.84	3.85
Primitive 1 (%)	73.4	53.1	42.3	39.1	35.8	35.2	33.2	31.9	32.13
Primitive 2 (%)	26.6	46.9	57.7	60.9	64.2	64.8	66.8	68.1	67.86
Computation Time (μs)	6.7	36.0	114.2	230.6	396.6	552.9	802.4	1044.24	1446.2

**Table 5:** Results of running the algorithm for different network sizes in the “*dense networks*” group.

Network Size (# nodes)	10	25	50	75	100	125	150	175	200
Avg # Hops	1.29	1.48	1.64	1.72	1.78	1.84	1.89	1.92	1.96
Avg # Neigh per node	6.34	11.58	16.84	21.27	24.39	26.93	29.49	31.18	33.51.
Without W nodes (%)	95.0	88.6	81.8	77.4	74.1	71.0	68.4	66.7	64.2
Primitives per Switch	2.88	4.86	6.51	7.70	8.50	9.04	9.4	10.06	10.27
Primitive 1 (%)	40.5	25.0	19.2	16.5	15.1	14.1	13.4	12.74	12.5
Primitive 2 (%)	59.5	75.0	80.8	83.5	84.9	85.9	86.6	87.2	87.5
Computation Time (μs)	20.5	81.3	230.8	449.9	720.1	1025.6	1356.8	1729.2	2159.8
